# Nanoindentation behavior of high entropy alloys with transformation-induced plasticity

**DOI:** 10.1038/s41598-019-43174-x

**Published:** 2019-04-29

**Authors:** S. Sinha, R. A. Mirshams, T. Wang, S. S. Nene, M. Frank, K. Liu, R. S. Mishra

**Affiliations:** 1 Center for Friction Stir Processing, Department of Materials Science and Engineering, University of North Texas, Denton, TX 76207 USA; 2 Department of Engineering Technology, College of Engineering, University of North Texas, Denton, TX 76207 USA

**Keywords:** Metals and alloys, Mechanical properties

## Abstract

Nanoindentation of three metastable dual-phase high entropy alloys (HEAs) was performed to obtain their inherent elastoplastic deformation responses. Excellent combination of hardness and elastic modulus in as-cast condition confirmed that, their inherently higher strength compared to other HEAs reported in literature, can be attributed to alloy chemistry induced phase stability. Further, hardness of 8.28 GPa combined with modulus of 221.8 GPa was obtained in Fe-Mn-Co-Cr-Si-Cu HEA by annealing the as-cast material, which is the best hardness-modulus combination obtained to date in HEAs from nanoindentation. On the other hand, although Fe-Mn-Co-Cr-Si HEA showed lower hardness and modulus than Fe-Mn-Co-Cr-Si-Al and Fe-Mn-Co-Cr-Si-Cu HEAs, the former alloy exhibited the highest strain rate sensitivity, as determined from tests performed at five different strain rates. The three alloys also had subtle differences in incipient plasticity and elastoplastic behavior, while retaining similar levels of hardness; and nanoindentation response showed microstructural dependence in friction stir processed, annealed and tensile-deformed specimens. Thus, the study highlighted that while higher strength was achieved by designing a class of HEAs with similar composition, any of the individual alloys can be tuned to obtain enhanced properties.

## Introduction

Introducing transformation induced plasticity (TRIP) and twinning induced plasticity (TWIP) effects in high entropy alloys (HEAs) enabled development of HEAs with tunable compositions and microstructures and superior mechanical properties^1–3^. The underlying design strategy of how to control and use the γ (f.c.c.) to ε (h.c.p.) transformation in these alloys generated a lot of interest in transformative HEAs among researchers across the world. Recently, another category, including Ti-Zr-Hf-Nb-Ta and Nb-Hf-Zr-Ti HEAs that show phase transformation from b.c.c. to either h.c.p. or orthorhombic martensites during deformation, was reported^4,5^.

Our recent research confirmed that HEAs with TRIP (hereinafter referred to as TRIP-HEAs) offer an exciting domain of microstructural flexibility that can be exploited to obtain exceptional mechanical properties^6–8^. While tensile deformation behavior reveals overall material properties, local plastic deformation characteristics can be studied using techniques such as nanoindentation. Local deformation behavior at the nanoscale is important for establishing deformation micro-mechanisms across various length scales.

Nanoindentation is a technique that has been used widely to study deformation at the nanoscale in various materials^9–15^. Aspects of this technique that have been reported include the effect of indenter size and geometry on nanoindentation measurements^16–24^. Many studies also discussed the application of nanoindentation to investigate crack growth and fracture behavior^25–31^, incipient plasticity and elastoplastic deformation mechanisms^32–35^. Estimation of hardness and elastic modulus from nanoindentation was established by Oliver and Pharr^36^. Various methods for interpreting nanoindentation data were explained by Doerner and Nix^37^ and other researchers^16,38,39^. Most importantly, the advantages of nanoindentation are that the onset of plastic deformation and the regime of elastic-plastic transition can be captured in detail, and mechanisms at the nanoscale can be studied. The latter is of utmost importance in nanocrystalline materials and fine structures like the friction stir processed (FSP) TRIP-HEAs that we are currently studying. Since the incipient plasticity behavior of these TRIP-HEAs is not known, insight into nanoscale deformation mechanisms could provide the pathway to establish the relation between local deformation characteristics and overall deformation behavior. Revealing the inherent deformability of the γ (f.c.c.) and ε (h.c.p.) phases and establishing the connection between deformation mechanisms across length scale will enable prediction of the deformation behavior.

Here, we present nanoindentation behavior of CS-HEA^6^, Al-HEA^7^ and Cu-HEA^8,40^ — three TRIP-HEAs that have similar but not the same composition. In fact, Al-HEA and Cu-HEA were derived from the CS-HEA composition (nominal compositions presented later). The premise for studying these three alloys is that we can obtain the nanoindentation properties (hardness and elastic modulus) of TRIP-HEAs as a class of HEAs, yet capture the subtle differences that result from alloy chemistry-induced phase stability. To achieve this objective, and to elaborate the inherent incipient plasticity of these alloys, the comparison of nanoindentation properties is focused on as-cast materials. Additional objectives of this study are to obtain information about the intrinsic deformability of γ (f.c.c.) and ε (h.c.p.) phases, and to study nanoindentation behavior in processed and deformed conditions. A complete understanding of the latter topics requires rigorous study over time, since these TRIP-HEAs encompass a huge domain of microstructural variation. However, the present work aims to investigate the microstructural dependence of nanoindentation behavior. Therefore, some specific experiments on thermomechanically processed (FSP and annealed) and tensile deformed specimens are also included in this study.

## Results and Discussion

### Hardness and modulus of as-cast materials from nanoindentation

**Figure 1:**
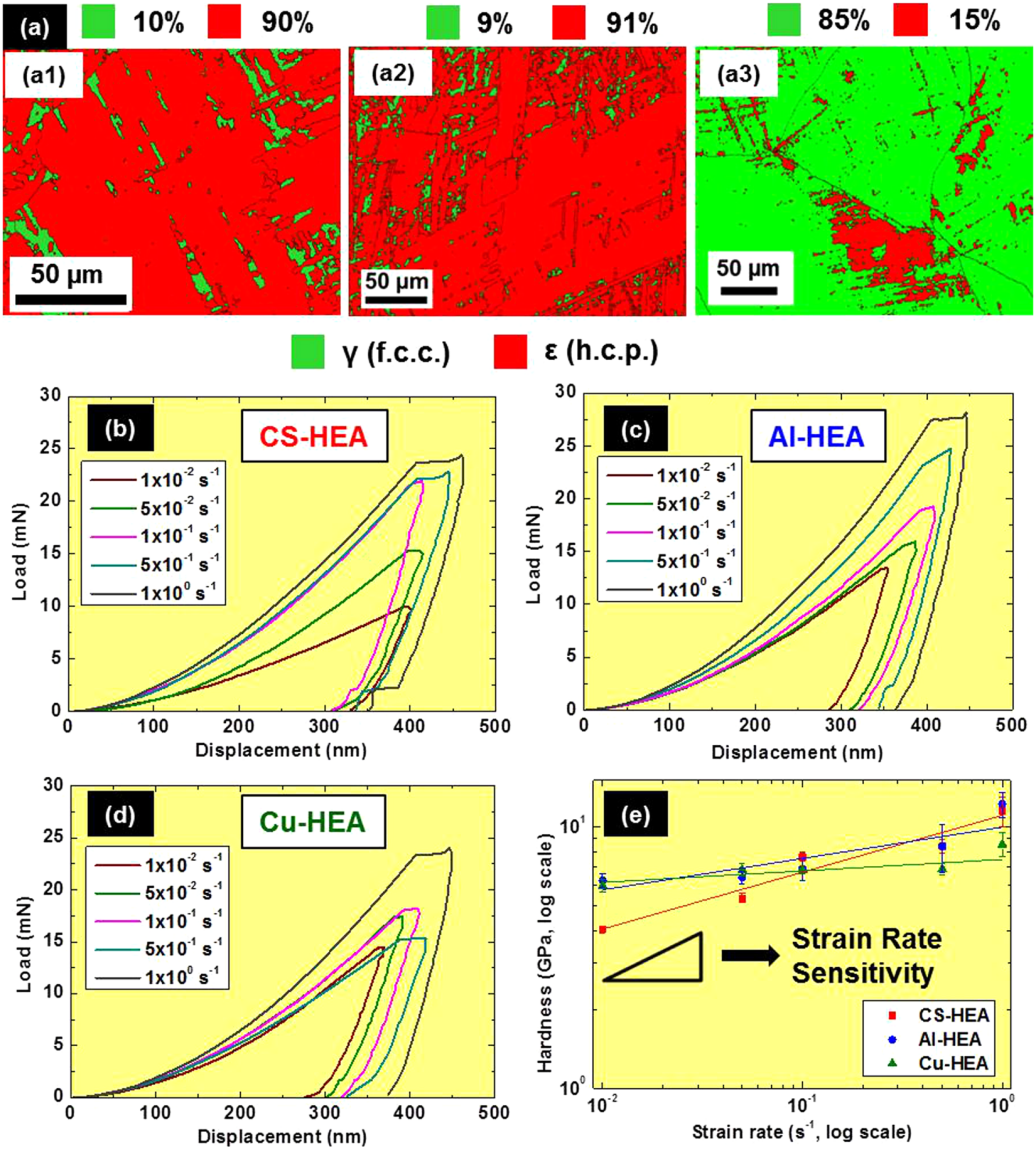
(**a**) EBSD phase maps showing microstructure of as-cast (a1) CS-HEA (a2) Al-HEA (a3) Cu-HEA; comparison of representative load-displacement curves obtained from nanoindentation tests at different strain rates for as-cast (**b**) CS-HEA (**c**) Al-HEA and (**d**) Cu-HEA; (**e**) hardness as a function of strain rate for the three HEAs for strain rate sensitivity calculation.

Hardness and elastic modulus values of as-cast CS-HEA, Al-HEA and Cu-HEA obtained from nanoindentation are presented in Table 1. Cu-HEA shows the highest hardness among the three alloys (6.83 GPa), while Al-HEA exhibits highest modulus of 205.7 GPa. The hardness-modulus combinations of Al-HEA and Cu-HEA are similar, with the elastic modulus being close to that of steel. However, CS-HEA has a lower hardness and modulus than Al-HEA and Cu-HEA. Recently, Li *et al*. studied the elastic modulus of five HEAs in their f.c.c. and h.c.p. phases^41^. They showed the variation of elastic modulus with alloy chemistry when all five compositions are in the same phase, as well as variation with f.c.c. or h.c.p. crystal structure for a particular alloy composition. Clearly, the elastic modulus of HEAs depends on both alloy chemistry as well as crystal structure. Due to this reason, in our as-cast materials, characteristic elastic modulus and hardness were obtained (irrespective of measured position) for each of our three alloys, indicating that the lattice distortion due to alloy chemistry is also an important factor in the as-cast materials (and not just f.c.c. or h.c.p. crystal structure). CS-HEA and Al-HEA are more metastable with ε (h.c.p.)-dominated microstructure in as-cast condition^6,7^ (Fig. 1(a1,a2)); while addition of Cu stabilizes γ (f.c.c.) phase and hence, Cu-HEA has γ (f.c.c.)-dominated microstructure^8,40^ (Fig. 1(a3)). Therefore, crystal structure of constituent phases is not the only factor influencing nanoindentation properties of HEAs. Since all the as-cast materials are coarse-grained (of the order of several microns), a polycrystalline effect (from grain boundaries) is also ruled out. Therefore, the difference in nanoindentation hardness/modulus is attributed to alloy chemistry. Alloying elements influence the dimensionless shear strength in HEAs^42^. Both Al-HEA and Cu-HEA are derived from CS-HEA composition, but adding an extra element (Al or Cu) altered the ideal shear strength, and resulted in differences in nanoindentation properties. The higher hardness/modulus from nanoindentation of Al-HEA and Cu-HEA suggests that both Al and Cu increase the ideal shear strength from the CS-HEA composition, while CS-HEA has lower hardness/modulus due to larger shear instability.

**Table 1:** Hardness and elastic modulus values of present TRIP-HEAs in as-cast condition.

Alloy	Hardness (GPa)	Modulus (GPa)
CS-HEA	5.33 ± 0.21	156.0 ± 9.0
Al-HEA	6.38 ± 0.36	205.7 ± 5.2
Cu-HEA	6.83 ± 0.39	192.6 ± 5.5

Note: Strain rate = 5 × 10^−2^ s^−1^.

From the standpoint of high entropy alloys, alloy chemistry is expected to have such a direct effect on nanoindentation behavior. Conceptually, each constituent element in a HEA is considered to be a solute embedded in the surrounding alloy matrix^43^. The fundamental source of strengthening is the interaction of dislocations with local compositional fluctuations. Therefore, intuitively, introducing a new alloying element could cause more local concentration fluctuations, thus triggering local modulus variation and associated dislocation line energy variation. Since nanoindentation behavior is strongly dependent on incipient dislocation activity, the alloy chemistry dependence of nanoindentation is more pronounced. On the other hand, the alloy chemistry effect on bulk deformation behavior is relatively indirect. In the case of micro/macro length scale deformation behavior, alloying additions influence the stability of the constituent phases in the microstructure, and in turn decide the overall mechanical response upon loading.

### Strain rate dependence of nanoindentation behavior of as-cast materials

The hardness/modulus values obtained from nanoindentation must, however, be considered in light of the sensitivity of nanoindentation behavior to loading rate^44^. The nanoindentation load-displacement curves of the three as-cast HEAs at different strain rates are shown in Fig. 1(b–d). Higher strain rate shifts the curve upwards, and the measured hardness also increases with increasing loading rate indicating the strain rate sensitivity of these alloys. The relative shifts in load-displacement curves suggest that CS-HEA has higher strain rate sensitivity as compared to Al-HEA and Cu-HEA.

Strain rate sensitivity (m) was calculated from nanoindentation results by taking the slope of the log-log plot of hardness versus strain rate (Fig. 1(e)). The ‘m’ values for CS-HEA, Al-HEA and Cu-HEA were 0.21 ± 0.04, 0.12 ± 0.04 and 0.04 ± 0.02, respectively. This calculation confirms that although CS-HEA has lower hardness/modulus than Al-HEA and Cu-HEA, it has higher strain rate sensitivity.

### Calculation of maximum shear stress

Nanoindentation load-displacement curves reflect dislocation activity because displacement bursts (leading to pop-ins in the curve) are related to activation of dislocation sources^45–47^. The displacement bursts become less prominent with increasing strain rate. Therefore, curves from low strain rate tests are more appropriate for analyzing dislocation activity. At the same time, the first displacement burst is independent of strain rate^45^. Load corresponding to the first displacement burst can be used to calculate maximum shear stress below the indenter^45^ and could correspond to the first nucleation of dislocations under the indenter^46,47^. However, various schools of thought have been offered^45^. Schuh mentioned that the pop-in event is most likely related to a heterogeneous process like dislocation source activation or multiplication, and undetectable dislocation activity may precede the pop-in^48^. Analysis of the nanoindentation load-displacement curve certainly provides valuable insight about incipient plasticity and elastoplastic deformation behavior of the material^49,50^. The displacement bursts (which reflect dislocation mediated plastic activity) are separated by elastic portions, so that the transition in elastic-plastic behavior can be predicted from the curves.

**Figure 2:**
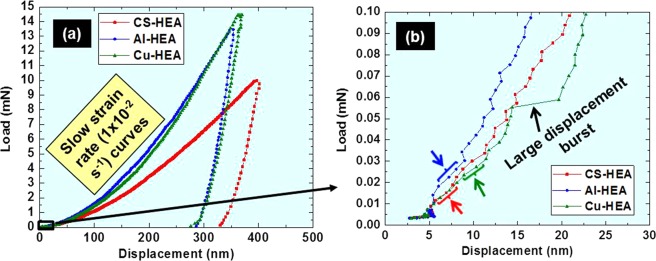
(**a**) Load-displacement curves from nanoindentation test (at 1 × 10^−2^ s^−1^ strain rate) of as-cast CS-HEA, Al-HEA and Cu-HEA, and (**b**) enlarged initial portion of the curve for calculation of maximum shear stress.

Load-displacement curves for the present TRIP-HEAs at low strain rate (1 × 10^−2^ s^−1^) and the enlarged initial parts of the curves are shown in Fig. 2(a,b), respectively. The loads corresponding to the first displacement burst are 0.018 mN, 0.026 mN and 0.024 mN for CS-HEA, Al-HEA and Cu-HEA, respectively. The region of the first displacement burst is marked by a bracket and arrow of the same color as the corresponding curve in Fig. 2(b), i.e. red for CS-HEA, blue for Al-HEA and green for Cu-HEA. The maximum shear stress under the indenter (τ_max_) was calculated using Eq. (1)^47^,

1 $${{\rm{\tau }}}_{{\rm{\max }}}=0.31{(\frac{6{{\rm{PE}}}_{{\rm{r}}}^{2}}{{{\rm{\pi }}}^{3}{{\rm{R}}}^{2}})}^{1/3},$$

and

2 $${{\rm{E}}}_{{\rm{r}}}={[\frac{1-{{\rm{\nu }}}_{{\rm{S}}}^{2}}{{{\rm{E}}}_{{\rm{S}}}}+\frac{1-{{\rm{\nu }}}_{{\rm{ind}}}^{2}}{{{\rm{E}}}_{{\rm{ind}}}}]}^{-1},$$

where E_r_ is the reduced modulus of the indenter/sample system; P is the load corresponding to the first displacement burst; R is the tip radius (50 nm); ν_s_ and ν_ind_ are the Poisson ratios of sample (0.3) and indenter (0.07), respectively; E_s_ and E_ind_ are the Young’s moduli of the sample and indenter (1000 GPa), respectively.

Maximum shear stress from the nanoindentation curve is an estimate of the theoretical shear strength of the material^45–47^. The values for τ_max_ for CS-HEA, Al-HEA and Cu-HEA of 8 GPa, 12.6 GPa and 11.5 GPa, respectively, project the difference in incipient plastic response of these TRIP-HEAs. At the same time, the frequency and nature of displacement bursts characterize the inherent elastoplastic behavior of the three alloys^45^. For example, in Fig. 2(b), the curve for Cu-HEA shows more pronounced displacement bursts, while the curves for CS-HEA and Al-HEA display short displacement bursts with elastic regions in between. Since the latter two alloys are h.c.p.-dominated, fewer slip systems are available compared to Cu-HEA, which is f.c.c.-dominated. Hence, large displacement bursts occur in Cu-HEA (one characteristically large burst is marked by black arrow in Fig. 2(b)); while the small displacement bursts in CS-HEA and Al-HEA indicate that dislocations are nucleated, but the resistance to dislocation motion suppresses overall dislocation activity. Again, between CS-HEA and Al-HEA (both h.c.p.-dominated), Al-HEA shows longer elastic regions between the characteristic small displacement bursts than CS-HEA. Thus, the greater inherent resistance to plastic deformation (or activation of dislocation sources) in Al-HEA is also consistent with the higher maximum shear stress (and hence theoretical shear strength) in Al-HEA than CS-HEA. This is due to higher lattice distortion effect in Al-HEA because Al is the largest atom in this alloy.

However, Li *et al*.^42^ discussed intrinsic ductility/brittleness based on the competition between ideal tensile strength (ITS), the propensity of brittle cleavage fracture; and ideal shear strength (ISS), the propensity of plastic flow, at the tip of a sharp crack. A lower ratio of ITS to ISS favors crack propagation rather than crack blunting, while a higher ratio indicates higher intrinsic ductility. CS-HEA showed higher yield stress (YS) in tension than Al-HEA in the as-cast condition and exceptional YS and ultimate tensile strength (UTS) after microstructural tailoring via FSP^6^. Subsequently, the design of Al-HEA^7^ enabled further improvement in strain hardening ability; wherein through suitable annealing treatment of as-FSP material, exceptional strength-ductility index was realized, while maintaining UTS close to CS-HEA. Therefore, the present nanoindentation study leads to the reasonable conclusion that while CS-HEA seems to possess higher intrinsic ductility, Al-HEA also has advantages on account of its intrinsic higher shear strength and incipient plasticity response.

During the early stages of deformation, more localized plastic deformation events occur in CS-HEA as compared to Al-HEA. On the other hand, Al-HEA also undergoes localized plastic deformation events but shows greater resistance to onset of plastic deformation. Despite these subtle differences, both alloys show similar intrinsic elastic-plastic behavior. Therefore, microstructural tuning of any of these alloys to obtain superior deformation response is possible.

### Microstructural dependence of nanoindentation behavior

The evolution of nanoindentation behavior with microstructural evolution from as-FSP condition to subsequent annealed and tensile deformed conditions was studied using a mini-tensile specimen of CS-HEA. The hardness and modulus in these three microstructural states were measured from nanoindentation and are shown in Table 2. However, note that the values obtained in this specific experiment must not be compared with the other values displayed for as-cast materials, because the specific tests were carried out on unmounted mini-tensile specimens glued on top of a cylindrical mount (unlike bigger mounted specimens for the as-cast materials, as described in Materials and Methods). Performing nanoindentation on unmounted tensile specimens glued to a mount for support as opposed to properly mounted specimens influences the stability of measurement conditions and hence, significantly different hardness/modulus values are obtained for the same specimen.

**Table 2:** Hardness and modulus of as-FSP, annealed and tensile deformed CS-HEA.

Specimen condition	Hardness (GPa)	Modulus (GPa)
As-FSP	4.1 ± 0.19	117.3 ± 3.9
Annealed	3.44 ± 0.41	103.4 ± 5.2
Tensile deformed	4.45 ± 0.39	131.0 ± 4.4

**Figure 3:**
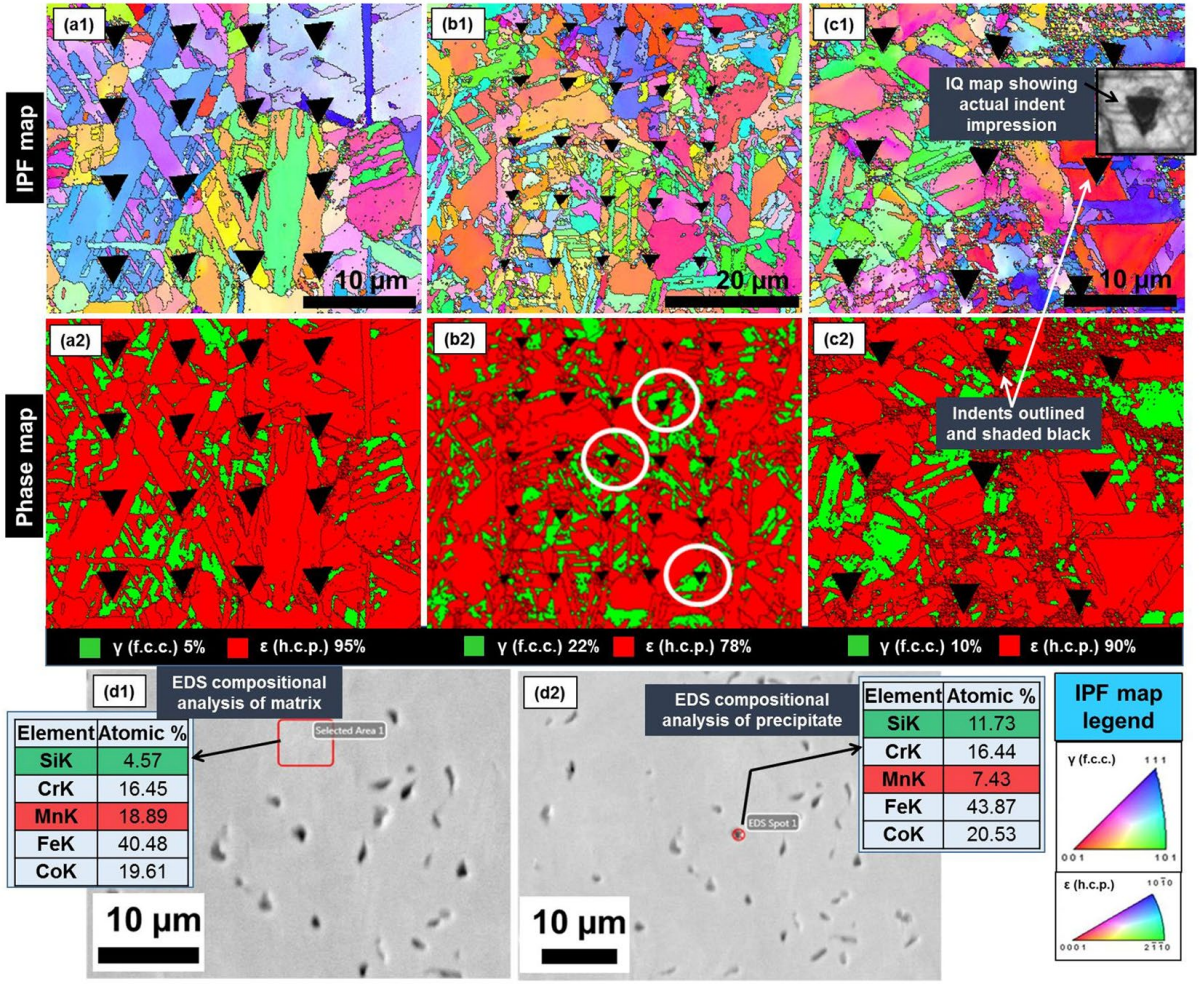
EBSD maps of nanoindented specimens of (**a1**,**a2**) as-FSP, (**b1**,**b2**) annealed; and (**c1**,**c2**) deformed CS-HEA (IQ map in inset in (**c1**) shows the original impression of an indent captured in EBSD, while in the IPF and phase maps, the indents are outlined and shaded black.); EDS compositional analysis of the annealed microstructure in the (**d1**) matrix and (**d2**) precipitate.

Figure 3(a–c) show the EBSD maps for the nanoindented specimens in the three conditions. The as-FSP microstructure (Fig. 3(a1,a2)) consisted of 5% γ (f.c.c.)–95% ε (h.c.p.) phases. The hardness was 4.1 ± 0.19 GPa and modulus was 117.3 ± 3.9 GPa. Although subsequent annealing was intended to transform to γ (f.c.c.)-dominated microstructure but could not be achieved in the present study, the annealed microstructure (Fig. 3(b1,b2)) showed increase in γ (f.c.c.) phase (22% γ–78% ε) with the appearance of precipitate-like features (Fig. 3(d1,d2)). EDS compositional analysis of the matrix and the particles confirmed that the latter are Si-rich (and Mn-depleted). In the annealed condition, hardness and modulus of the material were 3.33 ± 0.09 GPa and 104.6 ± 1.36 GPa, respectively. Thus, softening of the material upon annealing is reflected in the hardness/modulus. However, comparison of the indents on γ (f.c.c.) grains (circled in white in Fig. 3(b2)) with the indents on ε (h.c.p.) grains suggests that both phases in the dual-phase microstructure have similar properties. While a few earlier studies discussed nanoindentation of h.c.p. materials like Mg and Ti^51–53^, revealing the difference in inherent deformability characteristics of γ (f.c.c.) and ε (h.c.p.) phases in dual-phase TRIP-HEAs requires further investigation. Upon subsequent tensile deformation to failure, some γ to ε transformation occurred, with a resulting 10% γ–90% ε microstructure (Fig. 3(c1,c2)). This transformation was accompanied by increase in hardness (4.45 ± 0.39 GPa) and modulus (131 ± 4.4 GPa), and indicated hardening of the material during deformation. Thus, the microstructural dependence of nanoindentation behavior was captured. Therefore, the conclusion is that in addition to the intrinsic incipient plasticity resulting from alloying elements and crystal structure of phases, nanoscale deformation behavior is also responsive to thermomechanical processing and deformation; in this respect, local deformation response is similar to overall deformation behavior at micro- and macro-scales. The latter similarity across different length scales could be realized because the nanoindentation technique integrates the intrinsic properties of perfect material (like theoretical strength) with plasticity effects arising out of imperfections (defects and dislocation sources).

**Figure 4:**
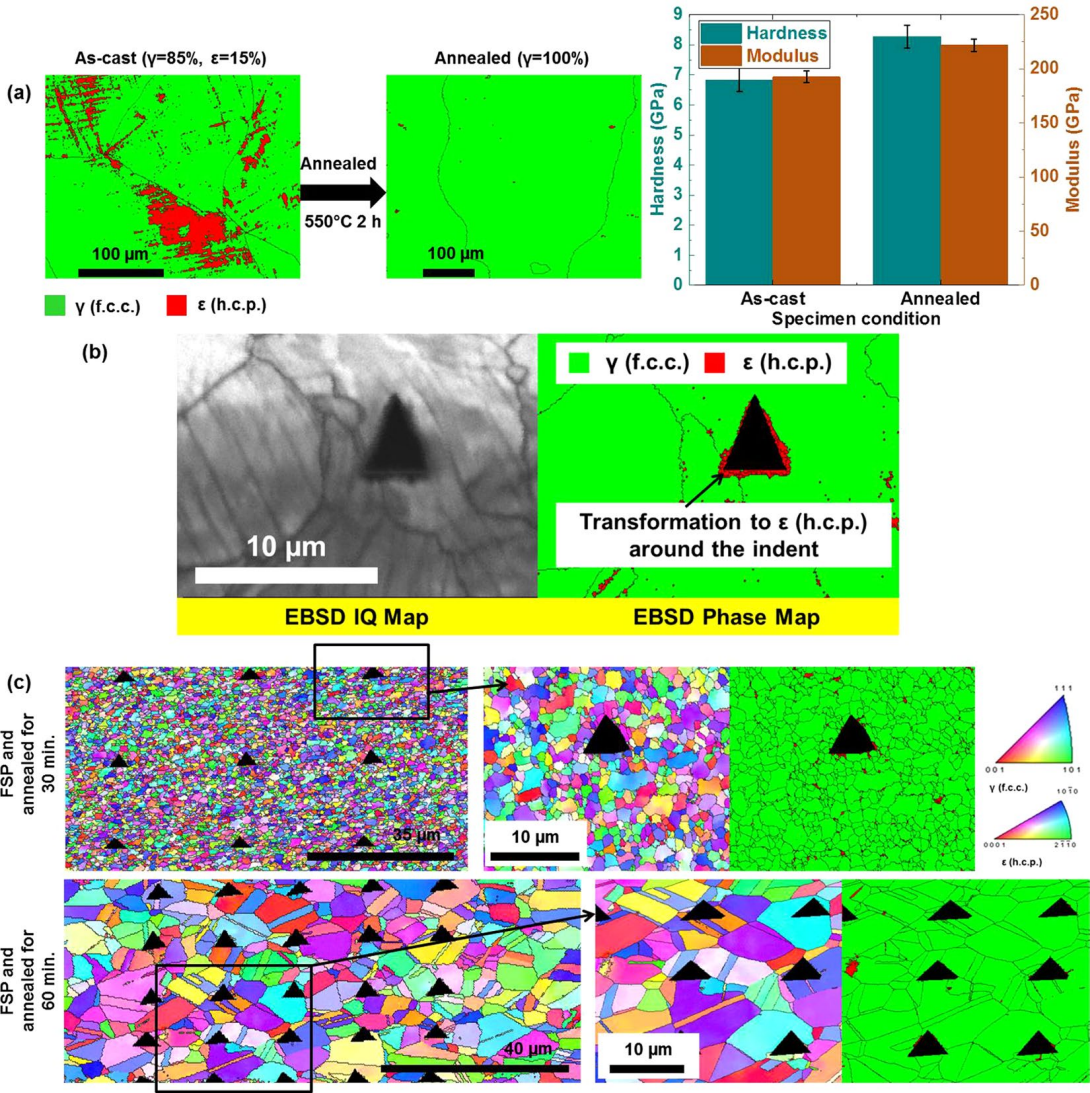
(**a)** EBSD phase maps and hardness-modulus plots as a function of specimen condition in as-cast and annealed Cu-HEA (**b**) EBSD IQ and phase maps showing transformation around indent in a coarse-grained f.c.c. microstructure of Cu-HEA (**c**) IPF maps and phase maps showing the effect of grain size in FSP-annealed Cu-HEA.

The previous specific experiment on as-FSP, annealed and deformed CS-HEA clearly indicated the microstructural dependence of nanoindentation response in TRIP-HEAs. Phase and grain evolution are important to determine the overall mechanical response (for example, tensile behavior) and more so for local plastic deformation characteristics, as studied in nanoindentation. Figure 4 elaborates further on the microstructural effects on nanoindentation behavior, this time using Cu-HEA as an example.

Figure 4(a) shows phase evolution with annealing in the as-cast Cu-HEA and the corresponding change in hardness and modulus from nanoindentation. Annealing led to transformation to 100% γ (f.c.c.) microstructure (from 85% γ–15% ε in the as-cast condition), accompanied by increase in both hardness and modulus. This is an interesting observation considering that annealing generally causes softening of the material (as also observed in the specific experiment on CS-HEA in Fig. 3). Hence, the effect of nanoindentation on phase transformation must be considered. Annealing leads to coarser, softer γ (f.c.c.) in as-cast Cu-HEA (Fig. 4(a)). Since this annealed γ (f.c.c.) is easier to plasticize, the probability of transformation to ε (h.c.p.) martensite beneath the indenter is higher in this condition. When martensitic transformation occurs due to nanoindentation, this accounts for the increased hardness measured by nanoindentation. Here, it must be mentioned that capturing the occurrence of phase transformation due to nanoindentation depends on the spread of the transformed zone. The transformed zone spreads to the surface and is clearly visible around the indent only in certain cases, especially when a higher indentation depth limit (1000–2000 nm) is used. Such an example in coarse grained f.c.c. microstructure of Cu-HEA is shown in Fig. 4(b), where the plastic zone around the indent is revealed to be ε (h.c.p.) martensite by the EBSD phase map, i.e. the transformed zone was large enough to spread to the surface around the indent. In some other cases, the transformed zone beneath the indenter was not captured in the 2D EBSD map of the surface and 3D imaging of the region beneath the indenter would be required to capture the transformed zone due to nanoindentation.

Figure 4(c) shows another example of the effect of grain size from FSP and annealed specimens of Cu-HEA. Again, note that these results illustrate an example of microstructural dependence and should not be compared with other hardness values reported elsewhere in the paper because unmounted specimens were used here. The EBSD IPF maps and phase maps (with nanoindents outlined and shaded in black) in Fig. 4(c) confirm that due to finer grain size in the 30 min. annealed specimen than the 60 min. annealed specimen, each nanoindent covered multiple grains in the former but only one or two grains in the latter. More importantly, each indent fell on multiple intersecting grain boundaries on the 30 min. annealed specimen; while, indents in the 60 min. annealed specimen were mostly in the grain interior or at most partly on the grain boundary between two grains. Thus, the polycrystalline effect (effect of grain boundaries) influences hardness, since indenting on the grain boundaries is harder than the grain interior. As a result, higher hardness was obtained in the 30 min. annealed specimen (4.06 ± 0.52 GPa) than in the 60 min. annealed specimen (3.5 ± 0.16 GPa). Also, elastic modulus was higher in the 30 min. annealed specimen (142 ± 11.1 GPa) than in the 60 min. annealed specimen (126.8 ± 4.2 GPa).

Finally, worth mentioning is that nanoindentation behavior is also influenced by crystallographic texture effects^54,55^ and interfaces other than grain boundaries (e.g., phase and twin boundaries), although these aspects were not studied in detail in the present study.

### Comparison of present TRIP-HEAs with literature

**Figure 5:**
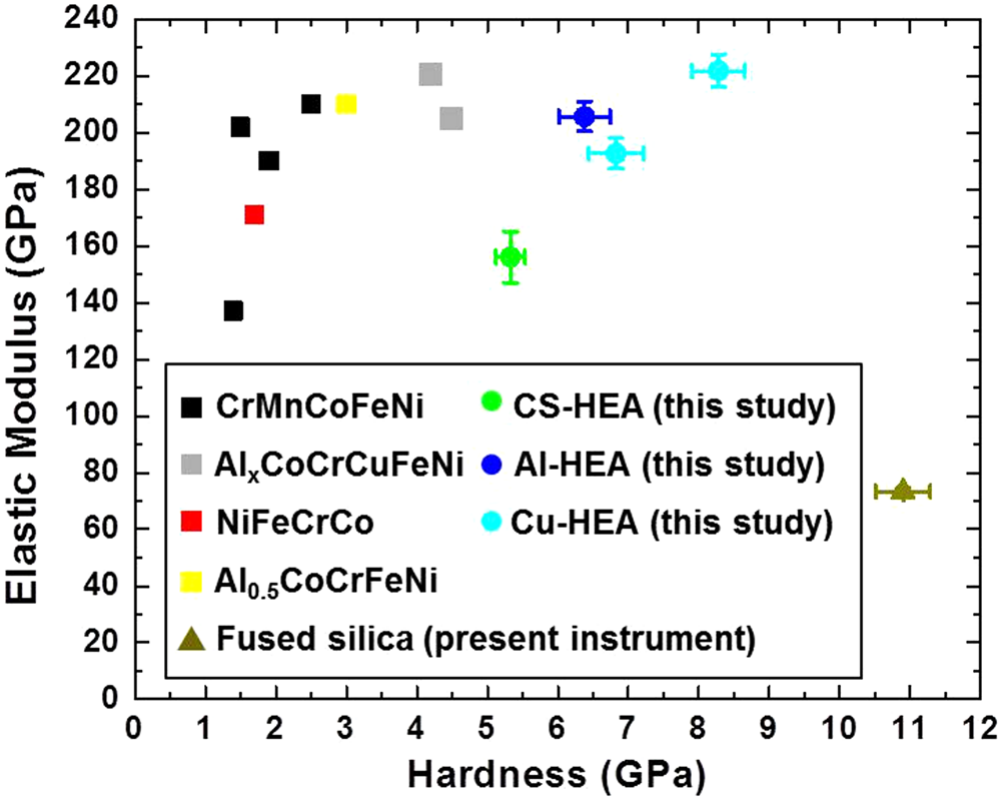
Comparison of present TRIP-HEAs with literature^57–63^; properties of fused silica measured using the present instrument are included for reference.

Figure 5 compares the elastic modulus-hardness combination of the present TRIP-HEAs with nanoindentation results on HEAs reported in literature. The comparison with literature is based on studies performed at similar strain rate (with present experiments performed at a strain rate of 5 × 10^−2^ s^−1^), to maintain consistency with present results. Clearly, all three TRIP-HEAs show higher hardness compared to other reported HEAs. However, Al-HEA and Cu-HEA also exhibit excellent elastic modulus-hardness combinations (around 200 GPa modulus with 7 GPa hardness). Further, 221.8 GPa modulus with 8.28 GPa hardness in Cu-HEA could be realized by annealing the as-cast material, which to date is the best modulus-hardness combination obtained in HEAs from nanoindentation. The modulus of CS-HEA was lower than Al-HEA and Cu-HEA. However, CS-HEA demonstrated the highest strain rate sensitivity (Fig. 1(e)) and seemed to possess higher intrinsic ductility. Al-HEA also evinced sustained elastoplastic behavior with greater shear strength. Therefore, clearly, the intrinsic elastic-plastic behavior of these alloys is interesting. The excellent tensile response of these alloys (depending on thermo-mechanical processing) has been reported^6,7^. While the potential of these TRIP-HEAs as candidates for structural materials was already established from the earlier studies, the remarkable deformation behavior of these alloys at the local (nano) scale is evident from the present study, and strengthens the hypothesis that the scope for tuning these alloys to obtain enhanced properties is immense.

## Conclusions

Nanoindentation study of the three TRIP-HEAs in the present investigation revealed that each of these alloys offers unique advantages with respect to nanoscale deformation behavior. Al-HEA and Cu-HEA exhibited higher hardness-modulus combinations than other reported HEAs. By annealing the as-cast Cu-HEA, we were able to obtain exceptional properties of 8.28 GPa hardness, with 221.8 GPa modulus. CS-HEA also demonstrated higher hardness than other HEAs, although its elastic modulus is low. However, CS-HEA proved to have the highest strain rate sensitivity. CS-HEA and Al-HEA showed similar elastoplastic behavior. While CS-HEA probably has higher intrinsic ductility than Al-HEA, Al-HEA exhibited higher maximum shear strength. Specific nanoindentation comparison of as-FSP, annealed and deformed specimens in CS-HEA confirmed that the nanoscale deformation behavior not only is dependent on alloy chemistry and crystal structure, but it is also responsive to thermomechanical processing and deformed state; the latter characteristic confirmed microstructure dependence of deformation mechanisms across various length scales.

## Materials and Methods

### Materials

Nominal compositions (in at. %) of HEAs in the present study were Fe_40_Mn_20_Co_20_Cr_15_Si_5_ (CS-HEA), Fe_39_Mn_20_Co_20_Cr_15_Si_5_Al_1_ (Al-HEA) and Fe_38.5_Mn_20_Co_20_Cr_15_Si_5_Cu_1.5_ (Cu-HEA). The alloys were produced by vacuum arc-casting in a cold copper crucible, using pure metals and ingot dimensions of 300 × 100 × 6 mm^3^. The chamber was backfilled with argon to 1 atm. prior to each melt.

### Friction stir processing (FSP), annealing and tensile deformation

Single-pass FSP of as-cast CS-HEA and double-pass (two overlapping runs) FSP of Cu-HEA were performed using a W-Re tool, with the parameters specified in Table 3. Tool dimensions were 12 mm shoulder diameter with tapered pin, 7.5 mm root diameter, 6 mm pin tip diameter and 3.5 mm pin length.

**Table 3:** Friction stir processing parameters used in the present study.

Alloy	Processing condition	Tool rotation rate (RPM)	Traverse speed (mm/min)	Plunge depth (mm)	Tilt angle (°)
CS-HEA	Pass 1	350	50.8	3.65	2
Cu-HEA	Pass 1	350	50.8	3.65	2
	Pass 2	150	50.8	3.70	2

A flat rectangular dog bone-shaped mini-tensile specimen with gage length 5 mm, width 1.25 mm, and thickness 1 mm was machined from 1 mm below the surface from the stirred region of the as-FSP CS-HEA material using a mini computer numerical control (CNC) machine. This mini-tensile specimen was used for nanoindentation in as-FSP and subsequent annealed and tensile deformed conditions.

Annealing of the as-FSP mini-tensile specimen of CS-HEA (showing ε (h.c.p.) microstructure) was performed at 850 °C for 30 min. with the objective of obtaining γ (f.c.c.) in the microstructure. Subsequent room temperature tensile test to failure of the annealed specimen (intended to transform back to fully ε (h.c.p.) microstructure) was done in a mini-tensile tester at an initial strain rate of 10^−3^ s^−1^. Annealing of as-cast Cu-HEA was performed at 550 °C for 120 min. For as-FSP Cu-HEA specimen, two-step annealing was conducted. First, annealing at 900 °C for 5 min. was done to obtain γ (f.c.c.) matrix supersaturated with Cu. Subsequently, annealing at 500 °C was done for 30 min. and 60 min. to precipitate out Cu. The specimens were quenched in water after each annealing step.

### Nanoindentation

Nanoindentation of TRIP-HEAs was performed using a XP-213 nanoindenter with a standard Berkovich tip with radius 50 nm and ϕ and β angles of 65.3° and 12.95°, respectively. The instrument was calibrated using a standard fused silica specimen. For as-cast materials (CS-HEA, Al-HEA and Cu-HEA), each test involved a 3 × 3 array of indents with spacing of 10 µm in ‘x’ and ‘y’ directions. The indentation depth limit for each indent was 400 nm, and average hardness from each test was obtained from the 200–300 nm depth range. Average values and standard deviation for each specimen were calculated based on all 9 indents. Strain rate was 5 × 10^−2^ s^−1^. Strain rate sensitivity (m) was calculated as a result of tests performed at strain rates of 1 × 10^−2^ s^−1^, 5 × 10^−2^ s^−1^, 1 × 10^−1^ s^−1^, 5 × 10^−1^ s^−1^ and 1 × 10^0^ s^−1^ and using the same parameters. The ‘m’ value was calculated by the procedure described by Song *et al*.^56^. For all these tests, a rectangular block specimen with dimensions at least 17 mm × 8 mm × 5 mm was used in the mounted condition. Nanoindentation was also performed on as-cast and annealed Cu-HEA specimen using similar specimens and identical testing parameters.

A specific nanoindentation experiment on CS-HEA compared the nanoindentation behavior of as-FSP, annealed and deformed microstructure using a rectangular mini-tensile specimen. The same mini-tensile specimen was used for testing in the three different conditions, and the specimen was glued on top of a cylindrical mount to perform the nanoindentation test. The same parameters were used for nanoindentation testing at a strain rate of 5 × 10^−2^ s^−1^. Results were based on multiple indents (9 or more).

FSP and annealed specimens of Cu-HEA were also tested by nanoindentation to obtain insight of the effect of γ (f.c.c.) grain size on hardness and modulus. Strain rate was 5 × 10^−2^ s^−1^. However, these tests were performed on mini-tensile specimens glued on top of a cylindrical mount. Also, the indentation depth limit for these tests was 1000 nm.

### Microstructural Characterization

Electron backscatter diffraction (EBSD) characterization of as-cast, as-FSP, annealed and tensile deformed specimens (before and after nanoindentation) was carried out on FEI Nova NanoSEM 230 with Hikari Super EBSD detector and an operating voltage of 20 kV. TEAM^TM^ EBSD analysis system was used for EBSD data acquisition, and TSL OIM Version 8 software was used for data analysis. Typical scan area and step size of EBSD acquisition were 75 µm × 60 µm and 0.2 µm, respectively.

## Data Availability

The raw/processed data required to reproduce these findings cannot be shared at this time as the data also forms part of an ongoing study.
